# PD-1/PD-L1 checkpoint inhibitor-induced encephalitis in patients with lung adenocarcinoma: a report of three cases

**DOI:** 10.1093/omcr/omae201

**Published:** 2025-03-20

**Authors:** Takayo Ota, Masaaki Terashima, Yukihiro Hamada, Shuichi Ueno, Shigeru Kawai, Noriko Tanaka, Kaoru Matsui, Noriyuki Masuda

**Affiliations:** Department of Breast Medical Oncology, Izumi City General Hospital, 4-5-1, Wake, Izumi, Osaka, 594-0073, Japan; Department of Medical Oncology, Izumi City General Hospital, 4-5-1, Wake, Izumi, Osaka, 594-0073, Japan; Department of Medical Oncology, Nara Kindai Faculty of Medicine, 1248-1, Otoda, Ikoma, Nara, 630-0293, Japan; Department of Neurology, Izumi City General Hospital, 4-5-1, Wake, Izumi, Osaka, 594-0073, Japan; Department of Neurology, Izumi City General Hospital, 4-5-1, Wake, Izumi, Osaka, 594-0073, Japan; Department of Neurology, Izumi City General Hospital, 4-5-1, Wake, Izumi, Osaka, 594-0073, Japan; Department of Radiology, Izumi City General Hospital, 4-5-1, Wake, Izumi, Osaka, 594-0073, Japan; Department of Breast Medical Oncology, Izumi City General Hospital, 4-5-1, Wake, Izumi, Osaka, 594-0073, Japan; Department of Respiratory Medicine, Izumi City General Hospital, 4-5-1, Wake, Izumi, Osaka, 594-0073, Japan; Department of Breast Medical Oncology, Izumi City General Hospital, 4-5-1, Wake, Izumi, Osaka, 594-0073, Japan; Department of Respiratory Medicine, Izumi City General Hospital, 4-5-1, Wake, Izumi, Osaka, 594-0073, Japan

**Keywords:** encephalitis, immune checkpoint inhibitors, PD-1/PD-L1 inhibitors, lung adenocarcinoma, brain metastases, cytomegalovirus

## Abstract

Immune checkpoint inhibitor (ICI)-induced encephalitis is rare. We present three cases of encephalitis associated with ICIs in lung adenocarcinoma patients. These patients presented with a variety of symptoms, but one of the common symptoms for all patients was loss of consciousness. All patients responded well to steroid treatment and survived longer than one month after the onset of symptoms. These cases highlight the difficulties in diagnosing encephalitis based only on clinical information, and timely management is important to improve survival. Opportunistic infections also have to be ruled out to diagnose ICI-induced encephalitis especially when brain metastases co-exist.

## Introduction

Immune checkpoint proteins regulate immune homeostasis and self-tolerance by maintaining a balance between stimulatory and inhibitory signals [1]. Immune checkpoint inhibitors (ICIs) activate exhausted T cells by inhibiting immune checkpoints, which enhance T-cell adaptive immunity against tumors and simultaneously cause immune-related adverse events (irAEs) across the organ system. The most used ICIs block two major immune checkpoint pathways: programmed death-1 (PD-1) or its ligand, programmed death ligand-1 (PD-L1), and cytotoxic T-lymphocyte-associated antigen (CTLA4). The incidence of irAEs caused by PD-1/PD-L1 inhibitors alone is estimated to be 64%–66% for all disease severity grades and 14%–21% for grade 3 or higher [2–4]. The incidence of neurological irAEs caused by ICIs in the range of 1%–12% [5, 6], and the incidence of irAEs caused by PD-1/PD-L1 inhibitors is greater than that caused by CTLA4. ICI-induced encephalitis (ICI-iE) is reported in 0.2%–0.5% of patients [7]. We report three cases of encephalitis associated with anti-PD-1/PD-L1 inhibitors in patients with lung adenocarcinoma (LUAD).

## Case report

### Case 1

**Figure 1:**
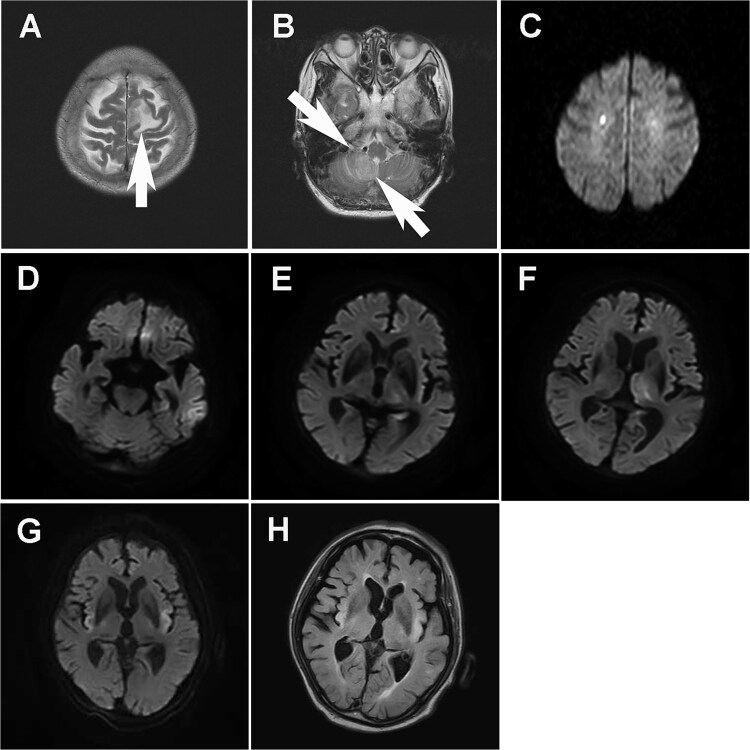
Brain MRI images. (A, B, C) case 1, (D, E, F) case 2, (G, H) case 3. (A, B) the edema surrounds the brain parenchyma in the right frontal lobe (arrow) and right cerebellar hemisphere (arrows). (C) DWI image shows acute lacunar infarction in the right frontal lobe. (D, E, F) DWI images show high signals in the (D) left parahippocampal gyrus and (E, F) left thalamus. (G) DWI image shows diffusion restriction, and (H) FLAIR image shows high intensity in both sides of the insular gyrus. The lesions are asymmetric.

In May 2017, a 75-year-old woman without a history of smoking was referred to our hospital with a 5-day history of lightheadedness and an unsteady posture on her feet. Brain magnetic resonance imaging (MRI) revealed tumors in the left frontal lobe and in the right cerebellar hemisphere (Fig. 1A and B). In addition, a chest computed tomography (CT) scan revealed a mass in the left upper lobe with multiple enlarged lymph nodes and osteolytic lesions in the lumbar vertebrae. While she underwent whole-brain radiotherapy under betamethasone treatment, a biopsy from bronchoscopy revealed adenocarcinoma. The cancer stage was determined to be cT2N3M1c, cStage IVB. The tumor proportion score (TPS) of programmed death ligand 1 (PD-L1) expression in the tissue was 90%. No variants were found in the tissue by molecular testing. Three days after the 2^nd^ cycle of pembrolizumab (200 mg) administration, she developed drowsiness and short-term memory loss (day 1). Blood tests, including leukocyte counts (5.7 × 10^9^/L) and C-reactive protein levels, were normal. Diffusion-weighted imaging (DWI) images of brain MRI showed a high-intensity signal, which was suspected to indicate acute lacuna infarction in the right frontal lobe (Fig. 1C). Anticoagulants were administered; however, her mental status decreased. On day 12, cerebral spinal fluid (CSF) analysis revealed a normal glucose level, a cell count of 51/μl with 94% monocyte, a protein level of 123.3 mg/dl (Table 1) and no malignant cells. Screening tests were negative for potential viral infections, except for both cytomegalovirus (CMV) IgG and IgM, which were detected by enzyme-linked immunosorbent assay in the serum and CSF. Beginning on day 17, ganciclovir was administered for 2 weeks; however, the level of consciousness gradually decreased. Electroencephalogram (EEG) revealed 4–6 Hz slow waves and no signs of status epilepticus. Brain MRI demonstrated no apparent changes compared to the previous MRI. Paraneoplastic antibodies were not tested. Repeated CSF analysis revealed a cell count of 24/μl and protein concentration of 79 mg/dl. Based on the history of pembrolizumab treatment and the clinical presentation, we suspected ICI-iE. On day 31, methyl-prednisolone pulse therapy (1 g/day for 3 days) was started, followed by treatment with tapered prednisolone for more than one month. Her consciousness gradually recovered, but her cognitive impairment did not improve.

**Table 1:** Summary of patient chractertistics and ICI-induced encephaitis.

	patient 1	patient 2	patient 3
Age	75	84	83
Sex	female	female	male
Malignancy	NSCLC	NSCLC	NSCLC
brain metastases at diagnosis of NSCLC	yes	no	no
Onset of symptoms (days after start date of therapy)	25 days	301 days	332 days
Weeks after first dose (cycle)	pembrolizumab 4 weeks (2 cycles)	pembrolizumab 30 weeks (10 cycles) + atezolizumab 12 weeks (4 cycles)	pembrolizumab 47 weeks (16 cycles)
Clinical presentation	lethargy, decreased level of conciousness	agitation, decreased level of concoiusness	convulsion, decreased level of conciousness
Magnetic Resonance Imaging (MRI)	Figure 1A–C	Figure 1D–F	Figure 1G,H
Electroencephalogram (EEG)	normal (day 33)	epilepsy (day 10)	epilepsy (day8)
Cerebrospinal Fluid (CSF) Profile	(day 12) glucose 57 mg/dl, protein 123.3 mg/dl, cell number 17/ulX3 (94% monocytic), IL-6 NA	(day10) glucose 76 mg/dl, protein 35 mg/dl, cell number 3/ulX3 (66% monocytic), IL-6 NA	(day 2) glucose 106 mg/dl, protein 24 mg/dl, cell number 4/ulX3 (25% monocytic), IL-6 26.9 pg/ml (ECLIA)
Autoantibodies	NA	recoverin (+/−)	none
Treatment	acyclovir, ganciclovir, prednisolone 1 g 3 days, then tapered	prednisolone 0.5 g 3 days	prednisolone 1 g 3 days, prednisolone 1 g 5 days
Outcome	resolved	resolved	resolved
Follow-up period	15 weeks	11 weeks	8 weeks

NA: not asessed, NSCLC: non-small cell lung cancer.

### Case 2

In September 2019, an 84-year-old female who had a 15-pack-a-year smoking history with advanced lung cancer was admitted to our hospital to undergo a course of palliative radiation therapy to improve her right shoulder pain. She was diagnosed with LUAD in the right upper lobe. The cancer stage was cT3N2M1b, cStageIVA. The TPS of PD-L1 expression in the tissue was 70%. Molecular testing of the tissue was negative. She started to receive pembrolizumab (200 mg) in December 2018. In total, she received ten cycles of pembrolizumab and four cycles of atezolizumab (1200 mg). On day 3, she developed muscle weakness in her extremities, after which she went into a coma. After 30 min, she recovered consciousness. Brain MRI revealed no space-occupying lesions or hemorrhage. On day 4, she became delirious during the night. On day 5, she tried to lock herself in the bathroom. She was agitated and kept in the bathroom for 30 min. After 90 min, she became calm and took quetiapine (25 mg) following the instructions. Radiation therapy was discontinued. On day 9, she experienced complex partial seizures. The seizures stopped temporarily with diazepam (10 mg), but her consciousness did not recover. DWI images of brain MRI showed high signals in the left thalamus and parahippocampal gyrus (Fig. 1D and F), indicating seizures or encephalitis. CSF analysis revealed a normal glucose level, a cell count of 9/μl, a protein concentration of 35 mg/dl, and no malignant cells (Table 1). Screening tests for viral infections were not conducted. The autoimmune workup was negative for anti-nuclear antibodies and rheumatoid factor. A serological paraneoplastic neurologic syndrome antibody panel (AMPH, CV2, PNMA2, Ri, Yo, Hu, SOX1, titin, zic4m GAD65, Tr, BML Inc. Tokyo) was negative, except for recoverin (+/−). EEG demonstrated myoclonus epilepsy. We suspected that the epilepsy was associated with ICIs. While continuing antiepileptic treatment, on day 11, methylprednisolone pulse therapy (500 mg/day for 3 days) followed by tapered steroids was administered. After 7 days, she gradually regained consciousness, and her seizures stopped without the use of anticonvulsant medications.

### Case 3

In August 2021, an 82-year-old male who had a 75-pack-a-year smoking history with advanced lung cancer was found by his wife lying on the ground at home at 10:30 am. Because of her dementia, she left him until 4:30 pm when his son found the patient. The patient lost consciousness with urinary incontinence. In August 2020, he was diagnosed with LUAD in the left upper lobe with pleuritis carcinomatosa. The cancer stage was cT1bN0M1a, cStageIVA. The TPS of PD-L1 expression in the tissue was 1%–49%. Molecular testing of the tissue was negative. He received 16 cycles of pembrolizumab (200 mg). When he came to our emergency department, his Glasgow Coma Scale (GCS) score was 13/15. His physical examination revealed spasms such as tic disorder on his face and slurred speech. On day 2, his level of consciousness decreased to a GCS score of 3/15. His vital signs were stable, and his blood glucose level was 163 mg/dl. Physical examination revealed spasms on both sides of the face and ocular deviation to the left. He was intubated and transferred to the intensive care unit (ICU). DWI images of brain MRI showed diffusion restriction, and fluid-attenuated inversion recovery (FLAIR) images showed high intensity in both sides of the insular gyrus (Fig. 1G and H). CSF analysis revealed a high glucose level, a cell count of 12/μl with 25% monocytes, a protein concentration of 24 mg/dl (Table 1), and no malignant cells. The autoimmune workup was positive for anti-nuclear (1:640) antibodies. A serological paraneoplastic neurologic syndrome antibody panel was negative (BML Inc. Tokyo). The history of pembrolizumab administration, the clinical presentation, the IL-6 concentration in CSF, and the brain MRI image, implying ICI-iE Starting on day 2, methyl-prednisolone pulse therapy (1 g/day for 3 days) was administered, together with acyclovir. On day 4, the spasm on his face subsided, and ocular deviation improved. On day 5, spasms were observed at the orbicularis oculi muscle in response to stimuli, and levetiracetam (1 g/day) was started; however, due to bradycardia, the treatment was stopped. On day 6, he was extubated. On day 7, EEG showed 3 Hz paroxysmal oculomotor discharges, indicating epilepsy. Screening tests were negative for potential viral infections. Starting on day 7, another methyl-prednisolone pulse therapy (1 g/day for 5 days) with levetiracetam (1 g/day) and valproic acid (800 mg/day) was administered. On day 10, the facial spasms disappeared. Afterward, the patient did not experience seizure relapse.

## Discussion

Neurological irAEs present with a wide variety of symptoms involving both the central nervous system (CNS) and the peripheral nervous system (PNS) [8]. CNS irAEs are less frequent than PNS irAEs. The mortality rate for CNS irAEs is believed to be relatively high [9]; however, a recent systematic review revealed that ICI-iE has favorable outcomes with rare fatal events [10].

The presentation of ICI-iE is nonspecific, which makes it difficult to recognize and diagnose. The most common presentations are altered mental status, focal CNS deficits, psychiatric symptoms, and seizures [7]. All three present cases demonstrated a decreased level of consciousness with a history of ICI administration; therefore, we suspected ICI-iE. Case 2 showed multiple neurological symptoms, possibly because we administered two different ICIs. The symptom onset of encephalitis is subacute (less than 3 months) [7]. In our cases, the onset was late, except case 1, which had brain metastases and was treated with radiotherapy. Disruption of the blood–brain barrier by brain metastases might trigger early ICI-iE; however, the onset of case 1 was in the general range.

ICI-iE is categorized into three groups according to their clinical features and presence of CNS inflammatory changes [11]: first, those with well-defined encephalitis accompanied by MRI abnormalities [12]; second, those with encephalitis with CNS inflammatory changes (CSF pleocytosis or new brain MRI abnormalities) without antibodies; and third, those with encephalitis without CNS inflammatory changes without antibodies. In their study, patients with ICI-iE without inflammatory changes had a high risk of mortality, and it was difficult for patients to respond to corticosteroid therapy. One-fifth of the patients died within one month after symptom onset. According to their classification, all our patients were classified into the 2^nd^ group and responded well to steroids, and their survival duration was longer than one month. Prompt recognition of the disease and early treatment are essential for managing ICI-iE because they prevent rapid T-cell-mediated neuronal loss [13].

To diagnose ICI-iE, opportunistic infectious etiologies, such as CMV infections, must be excluded. The incidence of CMV infection in the ICI-treated population is 0.3%–7.7% [14, 15]. CMV infection during ICI treatment can occur under the following three conditions: (i) CMV infection is a bystander, (ii) preceding immunosuppressive treatments for irAEs or other reasons reactivate latent CMV, and (iii) ICI triggers localized CMV reactivation through dysregulated inflammation [16]. CMV infection often occurs in organs affected by irAEs. In our case 1, precedent steroid treatment for brain metastasis may have reactivated latent CMV. Alternatively, ICI-iE may have triggered CMV infection. To date, no incidence rates of CMV infection have been reported for patients with ICI-iE.

In conclusion, we reported three cases of ICI-iE in NSCLC patients. The diagnosis of ICI-iE should be made by suspecting ICI-iE from the clinical presentations with the history of ICI administration. Opportunistic infections, such as cytomegalovirus, should be considered as one of differential diagnoses especially when brain metastases were present.
